# Excised leaves show limited and species-specific effects on photosynthetic parameters across crop functional types

**DOI:** 10.1093/jxb/erad319

**Published:** 2023-08-11

**Authors:** John N Ferguson, Tamanna Jithesh, Tracy Lawson, Johannes Kromdijk

**Affiliations:** Department of Plant Sciences, University of Cambridge, Cambridge, Cambridgeshire, CB2 3EA, UK; School of Life Sciences, University of Essex, Wivenhoe Park, Colchester, Essex CO4 3SQ, UK; Department of Plant Sciences, University of Cambridge, Cambridge, Cambridgeshire, CB2 3EA, UK; School of Life Sciences, University of Essex, Wivenhoe Park, Colchester, Essex CO4 3SQ, UK; Department of Plant Sciences, University of Cambridge, Cambridge, Cambridgeshire, CB2 3EA, UK; Institute for Genomic Biology, University of Illinois at Urbana-Champaign, Urbana, Illinois, 61801, USA; Universidade Nova de Lisboa, Portugal

**Keywords:** Barley, chlorophyll fluorescence, *Hordeum vulgare*, leaf excision, leaf reflectance, maize, photosynthesis, *Solanum lycopersicum*, tomato, *Zea mays*

## Abstract

Photosynthesis is increasingly becoming a recognized target for crop improvement. Phenotyping photosynthesis-related traits on field-grown material is a key bottleneck to progress here due to logistical barriers and short measurement days. Many studies attempt to overcome these challenges by phenotyping excised leaf material in the laboratory. To date there are no demonstrated examples of the representative nature of photosynthesis measurements performed on excised leaves relative to attached leaves in crops. Here, we tested whether standardized leaf excision on the day prior to phenotyping affected a range of common photosynthesis-related traits across crop functional types using tomato (C_3_ dicot), barley (C_3_ monocot), and maize (C_4_ monocot). Potentially constraining aspects of leaf physiology that could be predicted to impair photosynthesis in excised leaves, namely leaf water potential and abscisic acid accumulation, were not different between attached and excised leaves. We also observed non-significant differences in spectral reflectance and chlorophyll fluorescence traits between the treatments across the three species. However, we did observe some significant differences between traits associated with gas exchange and photosynthetic capacity across all three species. This study represents a useful reference for those who perform measurements of this nature and the differences reported should be considered in associated experimental design and statistical analyses.

## Teaser

Measurements of photosynthetic parameters across the functional types C_3_-dicot, C_3_-monocot, and C_4_-monocot in tomato, barley, and maize, respectively, demonstrate limited but important differences between excised and attached leaves.

## Introduction

The global demand for food is expected to double by the middle of this century. Human population growth means that the rate of increase in food demand outpaces the annual rate of increase in crop productivity (Zhu *et al.*, 2010; Kromdijk and Long, 2016; Lenaerts *et al.*, 2019). This mismatch highlights the necessity to adopt novel crop improvement targets to achieve food security (Gojon *et al.*, 2023). As the primary determinant of biomass accumulation, photosynthesis represents a sensible target to this end. Evidence from free-air CO_2_ enrichment (FACE) experiments has highlighted how increasing CO_2_ assimilation can lead to improved crop yields (Ainsworth and Long, 2021). Similarly, transgenic improvements to independent aspects of photosynthetic biochemistry have demonstrated that increasing carbon fixation is a realistic strategy for increasing yield (Driever *et al.*, 2017; Yoon *et al.*, 2020; De Souza *et al.*, 2022).

Targeting photosynthesis in crop improvement efforts necessitates the ability to screen many hundreds or thousands of genotypes for photosynthesis-associated traits. This is important for quantifying variation to incorporate in selection models. Additionally, it is necessary for facilitating forward genetics to identify novel genes or genetic regions underlying the variation to target through molecular approaches, such as marker-assisted breeding or direct manipulation through genome editing. This is challenging because the gold-standard methods through which many photosynthesis-associated traits are phenotyped are often logistically challenging to perform in field trial-like environments, and they are time consuming (Long and Bernacchi, 2003; Walker *et al.*, 2018). Standard methodologies, such as the commonly performed photosynthesis/CO_2_ response (*A*/*c*_i_) measurement used for modelling photosynthetic capacity, can take up to 45–60 min to perform per sample. This is problematic because it limits the number of measurements that can be performed per day. This is especially true in the field relative to controlled conditions, where diurnal weather effects on physiological patterns, such as decreasing leaf water potential, photosystem II efficiency, and chloroplastic inorganic phosphate concentration, can transiently affect photosynthetic activity and the length of the measurement day (Murchie *et al.*, 1999; Leakey *et al.*, 2006). Logistical challenges arise, for example, due to specialized equipment not being suitable for use within crop canopies and because of the lack of access to power supplies.

A potential means through which to overcome some of these confounding effects and logistical challenges is to excise leaf material from plants of interest and phenotype them in a more amenable and stable laboratory setting. Studies on woody species have shown that phenotyping traits associated with photosynthesis and reflectance on the leaves of excised branches generates data that is highly comparable to that generated through phenotyping leaves on branches still attached to the tree (Richardson and Berlyn, 2002; Miyazawa *et al.*, 2011; Akalusi *et al.*, 2021). Over the past decade, a similar approach has routinely been employed for multiple crop species (e.g. Driever *et al.*, 2014; Ferguson *et al.*, 2021; Montes *et al.*, 2022). Here, leaves, or tillers harbouring a leaf of interest, are excised from plants and the cut end is placed into water in an attempt to maintain hydraulic conductivity. For photo-physiological phenotyping, the maintenance of hydraulic efficiency is important for replacing water lost via transpiration (Müllers *et al.*, 2022), which is instrumental in keeping stomata open for CO_2_ uptake. It is possible that the demonstrable success of employing this approach with woody species is at least in part due to the presence of secondary xylem. Secondary xylem facilitates water transport, but it is also critical in protecting the primary xylem from mechanical damage (Miodek *et al.*, 2021), such as that which might ensue after branch excision. This presence of secondary xylem is a key anatomical difference that distinguishes the tree species where this approach has been demonstrated to work as effectively as *in situ* phenotyping and the herbaceous annual crop species for which this technique is now regularly employed. Due to this key difference (and others), it is plausible that the mechanistic basis that underpins the success of performing photosynthesis measurements on foliage of detached branches of woody species does not necessarily translate to herbaceous species. Consequently, a key knowledge gap exists here with respect to understanding the extent to which measurements made on excised leaves are representative of those made on attached leaves for crop species.

Addressing this knowledge gap is a high priority due to the increasing uptake of this approach for phenotyping photosynthesis in crop species. Variations on this technique have been reported in studies involving many important crops; however, we are not aware of any studies that employ this approach and show that the data they obtain are representative of what would have been obtained from intact material. Such information will be key for emphasizing the value of this approach. Maize is perhaps the species with which phenotyping of excised leaves has been most employed. For example, Markelz *et al.*, (2011) excised leaves from maize plants growing in a FACE experiment and under variable soil nitrogen availabilities to perform *A*/*c*_i_ response measurements to test the interactive effects of CO_2_ and N supply on photosynthetic capacity. Similarly, Liao *et al.*, (2022) phenotyped excised maize leaves from plants growing under full- and deficit-irrigation to perform *A*/*c*_i_ response measurements to test how water availability influenced photosynthetic capacity and stomatal limitations to photosynthesis. As well as phenotyping small numbers of maize genotypes to test environmental interactions (see also Leakey *et al.*, 2006, and Choquette *et al.*, 2019), this approach has also been employed to facilitate screening of large populations of maize for forward genetics purposes. For example, Xie *et al.*, (2021) used it to phenotype a mapping population consisting of 197 recombinant inbred lines for light-saturated gas exchange, thereby enabling the identification of the genetic regions underpinning variation in photosynthesis and associated traits. An identical approach has also been utilized for phenotyping genetic diversity in sorghum (Ferguson *et al.*, 2021). Here, it enabled by far the largest survey of genetic variation in photosynthesis to date, with over 800 genetically distinct sorghum varieties being characterized, which enabled genome- and transcriptome-wide association mapping (GWAS and TWAS) to pinpoint precise genes underlying the variation. Phenotyping photosynthesis of excised leaves has also been used in other C_4_ plants, such as *Miscanthus*, where the approach facilitated testing of the adaptation of photosynthesis to self-shading (Collison *et al.*, 2020), and sugarcane, where it facilitated testing of the response of photosynthesis to the application of sugars (McCormick *et al.*, 2008).

Whilst C_4_ crops typically have more physiologically robust leaves, measuring photosynthesis in excised material has also been performed in studies focusing on monocot and dicot C_3_ crops. For example, excised tillers have been used to phenotype flag-leaves for a suite of photosynthesis-related traits in a diversity panel of 64 wheat accessions at different developmental stages (Driever *et al.*, 2014; Carmo-Silva *et al.*, 2017). The same approach has also been used in barley where the leaf of interest was directly excised at its base rather than harvesting the entire tiller to test how direct nitrate feeding affected photosynthesis and sugar synthesis (Morcuende *et al.*, 2005). In rice, excised leaf material has also been used to measure chlorophyll fluorescence across different varieties to facilitate GWAS of fluorescence parameters (Ferguson *et al.*, 2020). Similar fluorescence experiments on excised leaf segments of wheat have provided some preliminary evidence for an effect of the time between excision and measurement, showing that a minimal waiting time might be needed to properly relate observations to differences in photosynthetic efficiency (McAusland *et al.*, 2019). To the best of our knowledge, utilization of excised leaf material in C_3_ dicots has so far been constrained to studies on soybean. For example, Morgan *et al.*, (2004) cut petioles of fully expanded leaves of soybean plants growing in an open-air elevated ozone experiment to perform chlorophyll fluorescence, *A*/*c*_i_, and photosynthesis light-response (*A*/*Q*) measurements to understand the photosynthetic response to ozone stress. The same technique was applied by Montes *et al.*, (2022) to perform *A*/*c*_i_ measurements and concurrent measurements of hyperspectral reflectance to build models for predicting photosynthetic capacity in the field and for facilitating GWAS.

Excision is likely to elicit an abscisic acid (ABA) biosynthesis response in leaves in a manner similar to that which promotes the stabilization of leaf water status (Beardsell and Cohen, 1975) and/or resistance to herbivory (Vos *et al.*, 2013, 2019, Preprint; Nguyen *et al.*, 2016). This is an important consideration here since ABA accumulation within leaves also serves to close stomatal apertures and reduce transpirational water loss in response to drought, which consequently impairs carbon uptake (Blatt, 2000; Schroeder *et al.*, 2001). In addition, there may well be a myriad of minor-to-major alterations of leaf properties that could affect the photosynthetic activity of excised leaves, for example changes to xylem tension, cell turgor, leaf water potential, and pigmentation (Govindjee *et al.*, 1981; Carter, 1991; Canny, 1997; Richardson and Berlyn, 2002; Tyree and Cochard, 2003). Given the existence of these potential pitfalls and because of our increasing reliance on performing photosynthesis measurements on excised leaf material in crop research, it is critical to understand whether leaf excision affects photosynthesis. In this study, we tested how standardized leaf excision on the day before phenotyping affected light-saturated photosynthesis, photosynthetic capacity, chlorophyll fluorescence, hyperspectral reflectance, ABA accumulation, and leaf water potential in comparison to leaves that remained attached to the plant. We employed reference genotypes of tomato, barley, and maize, which represent the main photosynthesis functional types of crops, namely C_3_ monocot, C_3_ dicot, and C_4_ monocot.

## Materials and methods

### Plant material and experimental design

The genotypes used in this study were tomato (*Solanum lycopersicum*, M82), barley (*Hordeum vulgare*, Golden Promise), and maize (*Zea mays*, B73). All plants were grown at the Plant Growth Facility of the University of Cambridge, UK. Seeds from all species were sown into modular trays before being transplanted into larger pots once the seedlings were established. Barley and tomato were grown in 1.5 l pots, whilst maize was grown in 6 l pots with two plants per plot. Barley was grown in a multipurpose compost (M3 Compost), tomato was grown in a seedling and cuttings compost (F2 Compost) and fertilized with a specialized tomato feed (Tomorite; all Levington Ltd., UK). Maize was grown in a 2:1 mix of these two compost types with added perlite. Tomato and maize were grown in the same growth room under a 14/10 h light/dark photoperiod with 600 µmol m^−2^ s^−1^ photosynthetically active radiation (PAR), at 28/20 °C and 65% relative humidity (RH). The growth room of the barley was set to a 16/8 h photoperiod with 400 µmol m^−2^ s^−1^ PAR, a constant temperature of 22 °C, and 60% RH.

On the day before measurements were taken, plants were transferred from the growth rooms to the nearby laboratory at 14.00 h. All plants were checked to be well-watered and left on the laboratory bench until 17.00 h, when those plants that were to be measured as excised were cut. For tomato, the youngest fully expanded leaf was cut at the petiole below the two leaflets adjacent to the terminal leaflet. The petiole was then immediately placed under water and recut before being transferred to a 15 ml falcon tube containing water. For barley, the youngest fully expanded leaf was excised at its base. The leaf was then recut under water just above the initial excision and transferred to a 15 ml falcon tube containing water. For maize, the youngest fully expanded leaf was excised at its base, recut under water just above the initial excision and transferred to a 50 ml falcon tube containing water. We ensured that all the tubes contained sufficient water so that the excision point was consistently submerged. The excised leaves were left on the same lab bench as their counterparts that remained attached to the plants. Leaf excision was performed on the day prior to phenotyping to ensure an appropriate time for ABA degradation and stabilization of leaf water potential.

On the measurement day, the excised and attached leaves were subjected to a series of parallel measurements commencing at 09.00 h, as illustrated in Supplementary Fig. S1 and detailed below. Tomato plants were measured 8 weeks after sowing across a 2 d period. Barley plants were measured across two separate 2 d periods one week apart, at 4 and 5 weeks after sowing. Maize plants were measured across two separate 2 d periods two weeks apart, at 6 and 8 weeks after sowing. Sampling date did not have a significant effect on any of the measured traits for any of the species, as determined through one-sample *t*-tests. Consequently, we combined the results from the different sampling times.

### Leaf-level gas exchange

Leaf-level gas exchange measurements were performed using two LI-COR 6400XT infra-red gas analysers fitted with 6400-40 fluorometer LED light sources (LI-COR Biosciences). To measure light-saturated gas exchange, conditions within the leaf chamber were set as follows: 25 °C block temperature; 400 µmol s^−1^ air flow; 65–75% RH; 400 µmol mol^−1^ reference CO_2_ concentration; 1500 µmol m^−2^ s^−1^ PAR. Once net photosynthetic CO_2_ assimilation (*A*_N_) and stomatal conductance (*g*_S_) were stable, the light-saturated rate of gas exchange was logged before initiating a CO_2_-response curve as described below. For each treatment, measurements were taken on 10–11 tomato plants, 7–8 barley, and 11–12 maize plants.

For the tomato and barley CO_2_-response curves, all conditions were kept as described above and gas exchange was measured at a series of reference CO_2_ concentrations as follows: 400, 300, 200, 100, 50, 400, 400, 700, 1000, 1300, and 1800 µmol mol^−1^. Measurements were logged when the gas exchange became stable, which took between 90–120 s at each point in the series. Measurements were taken on 8–10 tomato plants and 7–8 barley plants.

For the maize CO_2_-response curves, all conditions were again kept as described for the light-saturated gas exchange. The CO_2_ concentration was then increased in the following steps: 400, 600, 800, 1000, and 1250 µmol mol^−1^. The concentration was then returned to 400 µmol mol^−1^ and the values of *A*_N_ and *g*_s_ were allowed to re-stabilize, after which the CO_2_ concentration was reduced in the following sequence: 400, 300, 250, 200, 100, 75, and 25 µmol mol^−1^. Measurements were logged when the gas exchange became stable, which took between 60–90 s at each point in the series. Measurements were taken on 8–12 replicate plants. For all three species, CO_2_-response curves were determined in both the morning (AM) and afternoon (PM) (Supplementary Fig. S1).

For tomato and barley, The *A*_N_/*c*_i_ data from the response curves were fitted according to the FvCB model (Farquhar *et al.*, 1980) using the fitacis() function in the R package plantecophys (Duursma, 2015). This was achieved using the bilinear method to estimate the transition point. From this, estimates of the maximum rate of Rubisco carboxylation (*V*_cmax_) and the maximum rate of electron transport for RuBP regeneration (*J*_max_) on a *c*_i_ basis were obtained. For maize, the *A*_N_/*c*_i_ data from the response curves were fitted using a customized R function following von Caemmerer, (2000). Here, the initial relationship between *A*_N_ and *c*_i_ was used to estimate the maximum rate of carboxylation by PEPC (*V*_pmax_). In addition, the horizontal asymptote of a four-parameter non-rectangular hyperbola was used as an estimate of CO_2_- and light-saturated photosynthesis (*A*_max_).

### Hyperspectral reflectance

Hyperspectral reflectance measurements were performed using an ASD FieldSpec 4 Standard-Res Spectroradiometer equipped with a leaf-clip (Malvern Panalytical, Malvern, UK). The light source of the FieldSpec was allowed to warm up for 45 min prior to measurements. Hyperspectral reflectance was always measured on the same area of the leaf on which gas exchange was performed and always with the light source pointing towards the adaxial leaf surface. For each leaf, three technical replicates were performed and the reflectance at each wavelength was then averaged before further data analysis. The measurements were performed immediately following the CO_2_-response gas exchange measurements in the morning and afternoon, using the same biological replicates for each treatment and species combination.

### Chlorophyll fluorescence

Chlorophyll fluorescence measurements were performed using both the LICOR 6400XT leaf chamber fluorometer and using a closed chlorophyll fluorescence imaging system (FluorCam FC 800-C, PSI, Czech Republic). For both, a similar program was designed to measure the induction of non-photochemical quenching (NPQ) in response to actinic light and the subsequent relaxation of NPQ following the light being switched off.

Following the hyperspectral reflectance measurements conducted in the afternoon (Supplementary Fig. S1), a portion of the leaf was dark-adapted for 45 min using aluminium foil. The leaf was then clamped within the 6400XT leaf chamber, where conditions were as described above except that the light source was switched off. Following estimation of dark-adapted maximum fluorescence (*F*_m_), the light source was switched on to 1500 µmol m^−2^ s^−1^ PAR for 10 min and a series of 12 saturating pulses were applied to estimate maximal fluorescence (*F*_m_´) at different time-points. The light source was then switched off for 12 min and a further series of eight saturating pulses were performed again to estimate *F*_m_´. The NPQ at each point of measurement of chlorophyll fluorescence was then calculated as (*F*_m_–*F*_m_´)/*F*_m_´. Five biological repeats for each treatment and species combination were measured.

Samples for measurements of chlorophyll fluorescence in the imaging system were first analysed for leaf water potential using a Scholander pressure chamber (see below). Strips of ~3 × 5 cm were taken from the middle portion of the leaf for barley and maize, and from the terminal leaflet for tomato. The strips were arranged on damp filter paper and placed between pieces of non-reflective glass, as described by Ferguson *et al.*, (2020). The samples were then dark-adapted overnight (Supplementary Fig. S1) and measured for NPQ induction and relaxation within the closed chlorophyll fluorescence system following the same sequence of steps as described above for the 6400XT instrument. Measurements were taken on 11 biological replicates for tomato, eight for barley, and 12 for maize.

Using the NPQ data generated by both the leaf chamber fluorometer and the closed fluorescence system, separate exponential models were fitted to the light induction (Equation 1) and dark relaxation (Equation 2) components.


$$\begin{array}{l} NPQ=a\left(1-e^{-bt}\right) \end{array}$$ (1)

$$\begin{array}{l} NPQ=a\left(e^{-bt}\right)+c \end{array}$$ (2)

Where *a* represents the amplitude of the induction or relaxation response, *b* is the rate constant for the induction or relaxation of NPQ, *c* is an offset to account for a non-zero intercept (relaxation only), and *t* is the measurement time-point.

### Leaf water potential

Measurements of leaf water potential were performed using a Scholander pressure chamber (Model 600, PMS Instrument Company, OR, USA) following the manufacturer’s instructions. For the leaves still attached to the plants, a clean cut was made using a razor blade towards the base of the leaf for maize and barley or the petiole for tomato before immediately sealing the leaf into the chamber. For excised leaves, a new cut was made 3 cm above the cut from the previous day before immediately sealing the leaf into the chamber.

Leaf water potential measurements were made at the end of the measurement day between 18.00–18.30 h, after which the samples were excised for chlorophyll fluorescence measurements using the closed chlorophyll fluorescence system (Supplementary Fig. S1). Measurements were taken on 11 biological replicates for tomato, eight for barley, and eight or nine for maize.

### ABA analysis

Separate plants were grown for analysis of leaf ABA concentration under the same growth conditions as described above, and leaves were excised in the same manner. Barley and maize plants were measured 5 weeks after sowing and tomato plants were measured 8 weeks after sowing. Samples for ABA concentration measurements were taken at 10.00 h from leaves still attached to the plant and from leaves that had been excised at 17.00 h on the previous day. Each sample was taken from the middle portion of the leaf or from the terminal leaflet, taking care to avoid the midrib in barley and maize. The samples were immediately flash-frozen in liquid N and subsequently analysed for ABA concentration using a competitive ELISA kit (Phytodetek Immunoassay Kit for ABA, Agdia, Elkhart, IN, USA) following the manufacturer’s instructions. The area of each sample was measured before flash freezing, so that the ABA concentration could be expressed on a leaf area basis. Measurements were taken on six biological replicates for tomato, four for barley, and five for maize.

### Data processing and statistical analyses

All statistical analyses were performed within the R environment (www.r-project.org). All figures were generated using ggplot2 (Wickham, 2009) and gridExtra with some pre-processing in Affinity designer (Serif, Nottingham, UK).

We performed two-way repeated measures ANOVAs to test for significance of the treatment (excised or attached), the time of measurement (AM or PM), and their interaction for all gas exchange-associated traits. We performed one-sample *t*-tests to examine differences in leaf water potential and the traits associated with chlorophyll fluorescence between excised and attached leaves for each species independently. The chlorophyll fluorescence traits measured using the gas-exchange leaf chamber fluorometer and the closed chlorophyll fluorescence system were analysed independently because differences in sample conditioning, excitation wavelength, and detection optics precluded direct comparisons.

The asdreader R package was used for handling the hyperspectral reflectance data, following best practise outlined in Burnett *et al.*, (2021). The hyperspectral reflectance data were initially analysed through a spectra-wide approach where one-way ANOVAs were performed to test for differences in reflectance between the excised and attached leaves at each wavelength from 250–3500 nm. Separate tests were performed for each species and each time-point (i.e. AM and PM). We calculated a Bonferroni significance threshold (*α*=0.05) to avoid identification of false-positive results. It is worth noting that since there is covariation between wavelengths of a given spectra, this threshold is likely to be somewhat stringent. For visualization purposes, we performed a –log_10_ transformation of the *P*-value associated with each wavelength.

In addition, we used the same hyperspectral reflectance data to calculate multiple reflectance indices (Supplementary Table S1). We used these indices to perform repeated measures two-way ANOVAs separately for each species to test for effects due to treatment, time-point, and their interaction.

## Results

### Leaf water potential and accumulation of ABA

**Fig. 1.**
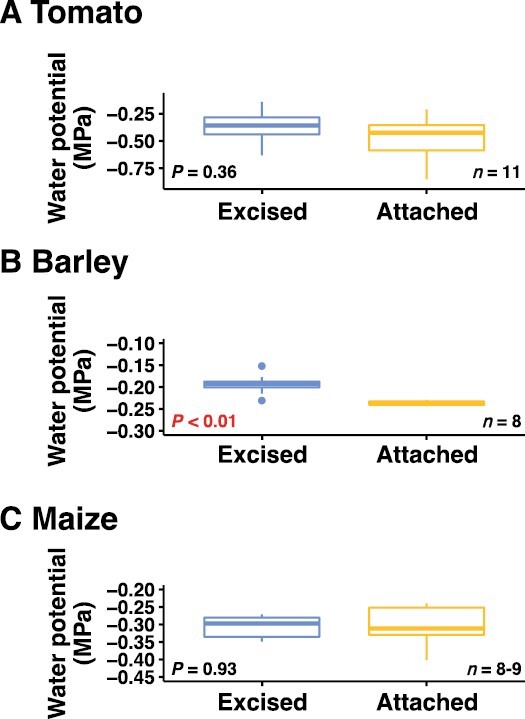
Effects of excision on leaf water potential. The boxplots show differences in leaf water potential between excised and attached leaves for (A) tomato, (B) barley, and (C) maize. Excised leaves were cut from the plant the day before measurement whilst attached leaves were cut immediately prior to measurement. The *P*-values were determined using one-sample *t*-tests.

Leaf water potential was determined at the end of the measurement day (Fig. 1; Supplementary Fig. S1). For tomato and maize, we did not observe a significant effect of leaf excision on leaf water potential; however, for barley the water potential of excised leaves (–0.193 MPa) showed a small but significant difference with that of leaves still attached to the plant (–0.237 MPa; Fig. 1B).

**Fig. 2.**
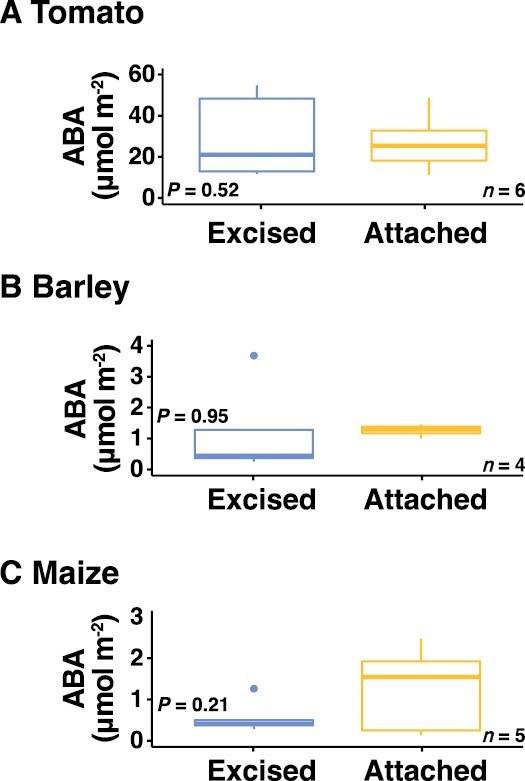
Effects of excision on leaf ABA concentration. The boxplots show differences in leaf ABA concentration between excised and attached leaves for (A) tomato, (B) barley, and (C) maize. Excised leaves were cut from the plant the day before measurement whilst attached leaves were sampled immediately prior to measurement. The *P*-values were determined using one-sample *t*-tests.

Leaf ABA concentration was measured in the morning after the excision treatment was carried out. For all three species, leaf excision did not result in any significant change in ABA accumulation compared to leaves that had remained attached to the plants (Fig. 2).

### Light-saturated gas exchange

**Fig. 3.**
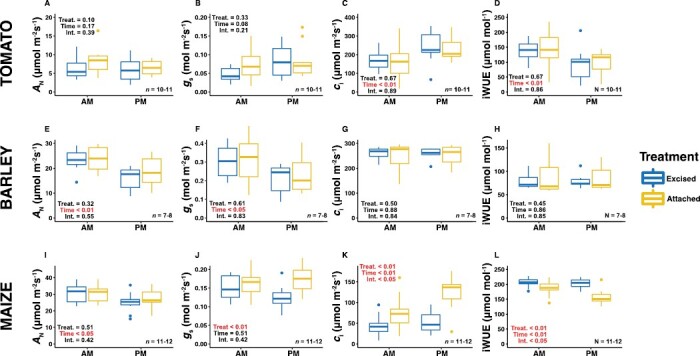
Effects of excision on leaf gas exchange parameters and intrinsic water use efficiency. Excised leaves were cut from the plant the day before measurement whilst attached leaves remained on the plant during measurement. Gas exchange was measured in the morning (AM) and afternoon (PM; see Supplementary Fig. S1). The boxplots show differences in light-saturated net photosynthesis (*A*_N_), stomatal conductance (*g*_s_), intracellular CO_2_ concentration (*c*_*i*_), and intrinsic water use efficiency (iWUE = *A*_N_/*g*_s_) for (A–D) tomato, (E–H) barley, and (I–L) maize. The *P*-values for the effects of treatment (Treat.), time, and their interaction (Int.) were determined using two-way ANOVA.

Light-saturated gas exchange was determined prior to the initiation of the *A*_N_/*c*_i_ response measurements in both the morning and the afternoon of the day after the leaf excision treatment (Supplementary Fig. S1. Across all three species, leaf excision did not have a significant effect on light-saturated *A*_N_ (Fig. 3). The timing of the measurement did not have a significant effect on *A*_N_ for tomato, but it did for barley and maize, with a reduction being observed in the afternoon independent of the treatment (Fig. 3A, E, I). Tomato also showed no significant effects for *g*_s_ (Fig. 3B). In barley, time had a significant effect on *g*_s_, with a reduction being observed in the afternoon similar to that seen for *A*_N_ (Fig. 3F). The excision treatment did have a significant effect on *g*_s_ for maize, with it being reduced in the excised leaves (Fig. 3J). Although the treatment × time interaction for *g*_s_ was not significant in maize, the difference between the excised and attached leaves did appear to increase in the afternoon. Barley showed no significant effects for *c*_i_ (Fig. 3g), whilst for tomato a significant time effect was observed with *c*_i_ increasing in the afternoon across both treatments (Fig. 3C). For maize, treatment, time, and the treatment×time interaction all had significant effects on *c*_i_ (Fig. 3K). Attached leaves showed higher *c*_i_ than excised leaves in both the morning and the afternoon, but the difference increased in the afternoon. The results for intrinsic water use efficiency (iWUE; the ratio of *A*_N_ to *g*_s_) mirrored those observed for *c*_i_ (Fig. 3D, H, L), such that it was significantly reduced in the afternoon for both tomato and maize, and it was significantly increased in the excised leaves of maize. For maize, it was notable that the differences in *c*_i_ and iWUE between the morning and afternoon were much greater in the attached leaves, and the values were more stable across the day in the excised leaves.

### Photosynthetic capacity

**Fig. 4.**
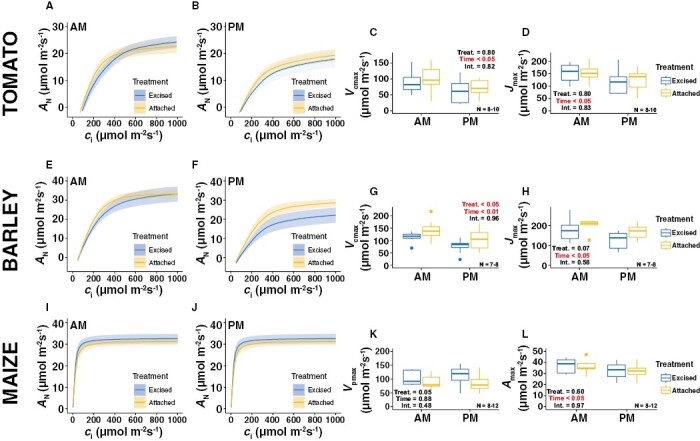
Effects of excision on leaf photosynthetic capacities modelled via photosynthesis/CO_2_ response curves. Excised leaves were cut from the plant the day before measurement whilst attached leaves remained on the plant during measurement. Gas exchange was measured in the morning (AM) and afternoon (PM; see Supplementary Fig. S1). Results are shown for (A–D) tomato, (E–H) barley, and (I–L) maize. (A, E, I) The response of light-saturated photosynthesis (*A*_N_) to increasing intracellular CO_2_ concentration (*c*_i_) in the morning and (B, F, J) in the afternoon. The lines represent the mean fit of the FvCB model for tomato and barley, and the mean fit of a four-parameter non-rectangular hyperbola for maize. The shaded areas represent the standard error of the mean. (C, G, K) The maximum carboxylation rates of (C, G) Rubisco (*V*_cmax_) for tomato and barley, and of (K) PEPc (*V*_pmax_) for maize. (D, H) The maximum rate of electron transport for RuBP regeneration (*J*_max_) for tomato and barley. (L) The light- and CO2-saturated rate of photosynthesis (*A*_max_) for maize. The *P*-values for the effects of treatment (Treat.), time, and their interaction (Int.) were determined using two-way ANOVA.

We modelled the *A*_N_/*c*_i_ response measurements to estimate the photosynthetic capacities of the three species in the morning and the afternoon (Fig. 4). Across all three, time had a significant effect on all the modelled parameters except *V*_pmax_ in maize (Fig. 4K). In all other cases, the parameters declined in the afternoon regardless of whether the leaf being measured was excised or remained attached to the plant (Fig. 4C, D, G, H, L). We only detected one significant treatment effect, with *V*_cmax_ being significantly reduced in excised barley leaves; however the reduction appeared to be relatively minor (Fig. 4G). In addition, the *P*-value associated with the treatment effect for *V*_pmax_ in maize was only marginally above 0.05 (Fig. 4K). Across all traits and species, we did not observe any significant interaction effect of treatment×time, suggesting that the observed effects were temporally consistent.

### Leaf reflectance

**Fig. 5.**
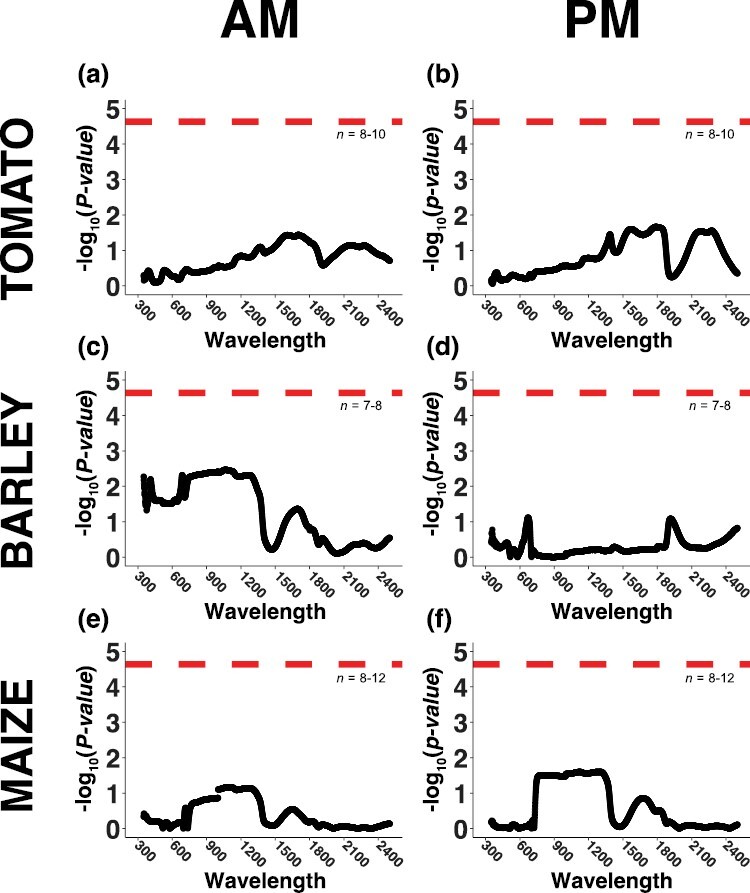
Effects of excision on leaf hyperspectral reflectance. Excised leaves were cut from the plant the day before measurement whilst attached leaves remained on the plant during measurement. Reflectance was measured in the morning (AM) and afternoon (PM; see Supplementary Fig. S1). (A, B) Tomato, (C, D) barley, and (E, F) maize. Each panel shows the -log_10_-transformed *P*-value from a one-way ANOVA comparing reflectance of attached and excised leaves at each incremental wavelength from 350–2500 nm. The dashed lines indicate a Bonferroni significance threshold of α=0.05.

Hyperspectral reflectance was measured following the determination of the *A*_N_/*c*_i_ responses in the morning and afternoon (Supplementary Figs S1, S2). None of the specific wavelengths showed any difference in reflectance between the excised and attached leaves in either the morning or the afternoon for any species according to the Bonferroni significance threshold (Fig. 5). Despite the lack of significant wavelength–treatment associations, it was still interesting to observe distinct species-specific patterns in the spectra-wide reflectance differences between the treatments, which can be interpreted based on the dominant factors that are known to control leaf reflectance across the hyperspectral range. The patterns of *P*-values across the spectral range were similar in the morning and afternoon for tomato (Fig. 5A, B) and maize (Fig. 5E, F); however, the patterns were distinctly different for barley (Fig. 5C, D). For tomato, the *P*-values showed a general trend of decreasing [inverse for –log_10_(*P*-value)] from 300 nm to 1800 nm before increasing between 1800 nm and 1900 nm. They then decreased to roughly what they were before the increase, before increasing again toward the end of the spectral range (Fig. 5A, B). This pattern suggests potential differences in leaf water status between the excised and attached tomato leaves. For maize, the pattern of *P*-values across the spectral range partially resembled the pattern of reflectance (Fig. 5E, F; Supplementary Fig. S2), with a broad peak of low *P*-values between 600 nm and 1400 nm and a narrower peak between 1500 nm and 1800 nm. The initial peak might reflect differences in leaf structure and the secondary peak is often associated with differences in leaf water status. For barley in the morning, the pattern of *P*-values across the spectral range was similar to that of maize reflecting potential effects of excision on leaf structure and water absorption (Fig. 5C, E). However, the initial portion of the range was also characterized by comparatively reduced *P*-values, especially around 400 nm (Fig. 5C), which might suggest an effect on the absorption of light by chlorophyll *b*. In the afternoon, the broad peak of low *P*-values between 600 nm and 1400 nm disappeared, but there were still minor peaks between 600 nm and 700 nm and between 1800 nm and 2000 nm (Fig. 5D), suggesting potential minor effects on chlorophyll absorption and water status, respectively.

Using the hyperspectral reflectance data (Supplementary Fig. S2), we also calculated reflectance indices related to nitrogen status, chlorophyll content, and leaf water status (Supplementary Table S1). We then examined how these were affected by leaf excision and time of measurement via two-way repeated measures ANOVA tests. Although this approach did find some significant differences, the observed effects were minor in all cases.

For tomato, we did not observe any significant treatment, time, or treatment×time interaction for any of the reflectance indices (Supplementary Fig. S3). For barley, we observed a significant time effect for the Datt Index, which is a proxy of total chlorophyll content, with higher values in the afternoon (Supplementary Fig. S4B). We also observed a significant interactive effect for barley for the moisture stress index (MSI), which is an inverted proxy for water content, with excised leaves having a higher MSI in the morning only, which suggests that their water content was reduced compared to attached leaves at this time-point (Supplementary Fig. S4D). For maize, the only significant effect observed was for treatment for the Datt Index, which was marginally higher in attached leaves compared to excised leaves, suggesting that the chlorophyll content was reduced following leaf excision (Fig. S5B).

### Chlorophyll fluorescence

**Fig. 6.**
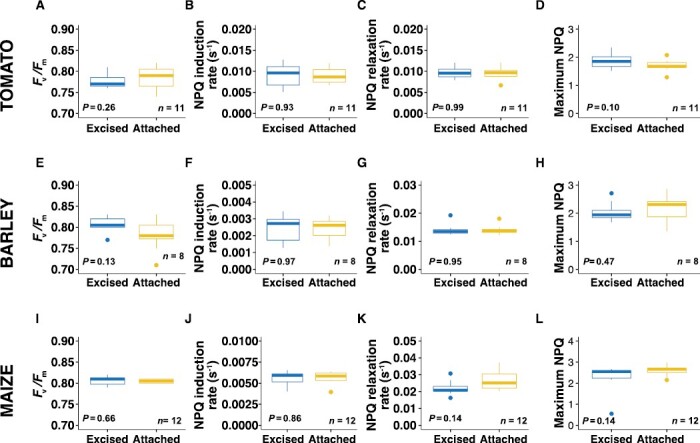
Effects of excision on leaf traits associated with chlorophyll fluorescence, as determined using a closed imaging system. Excised leaves were cut from the plant 2 d before measurement whilst attached leaves remained on the plant until being sampled for measurement (see Supplementary Fig. S1). (A–D) Tomato, (E–H) barley, and (I–L) maize. (A, E, I) Maximum efficiency of photosystem II (*F*_v_/*F*_m_), (B, F, J) the rate of induction of non-photochemical quenching (NPQ), (C, G, K) the rate of relaxation of NPQ, and (D, H, L) the maximum NPQ. The *P*-values were determined using one-sample *t*-tests. For results obtained 1 d after leaf excision using a leaf-chamber fluorometer see Supplementary Fig. S6.

Chlorophyll fluorescence was measured using both the leaf chamber fluorometer that was paired to the gas exchange platform and using a closed chlorophyll fluorescence imaging system on the following day (Supplementary Fig. S1). We tested for significant differences between samples taken from excised and attached leaves for measurements of *F*_v_/*F*_m_, the rates of induction and relaxation of NPQ (b_ind and b_rel, respectively), and maximum NPQ. For the samples measured using the closed chlorophyl fluorescence system, we did not observe any significant differences for any of these four parameters (Fig. 6). similar results were also obtained for these parameters with the leaf chamber fluorometer, expect for tomato b_ind, which was found to be significantly faster in attached leaves compared to excised leaves (Supplementary Fig. S6B). It was also noticeable that the *P*-values for *F*_v_/*F*_m_ (0.07) and maximum NPQ (0.09) were also close to the significance threshold, with attached leaves tending to have higher photosystem II maximum efficiency and reduced maximum NPQ (Supplementary Fig. S6A,D).

## Discussion

Screening variation in photosynthetic traits across large numbers of genotypes is a key requirement for targeting photosynthesis for crop improvement. However, such large-scale screening is fraught with logistical challenges, and these are commonly circumvented via phenotyping excised leaf material. A common limitation of these studies is the lack of evidence to demonstrate the representative nature of such phenotyping, which thus represents a key knowledge gap for this field. Hence, the principal aim of this study was to ascertain whether photosynthetic parameters measured on excised leaves of tomato, barley, and maize were representative of the same parameters measured on leaves that remained attached to the plants. There are at least three studies that have attempted to quantify potential differences in non-crop species. Using the evergreen tree species Japanese stone oak (*Lithocarpus edulis*), Miyazawa *et al.*, (2011) demonstrated that many photosynthetic parameters showed no significant differences when measured on excised leaves relative to attached leaves. The only trait that did demonstrate a significant difference was stomatal conductance, which was significantly higher in excised leaves. Despite this difference, the study showed that it was possible to use data from excised leaves to parametrize an ecophysiological model to reproduce diurnal patterns of *in situ* gas exchange. In a similar study, Akalusi *et al.*, (2021) showed that there were negligible differences in light-saturated rates of gas exchange and modelled estimates of photosynthetic capacity between excised and attached leaves of balsam fir (*Abies balsamea*). Moreover, the study also demonstrated that the non-significant differences carried through across the growing season. In a third study, Richardson and Berlyn (2002) focused on how spectral reflectance and chlorophyll fluorescence parameters were affected by leaf excision in four temperate tree species, and they found only minor differences at 12 h after excision. Furthermore, they demonstrated that with appropriate handling to maintain leaf water status, representative data could be obtained from excised leaves as late as 3 d post excision depending on the species. These studies therefore demonstrated the effectiveness of phenotyping traits related to photosynthesis on detached leaves of perennial woody species. As we noted in the Introduction, a similar approach is now commonly employed for many crop studies. The numerous differences that distinguish herbaceous annuals from trees suggest that the success of this approach might not carry over to crop species, and hence the rationale for our current study. Our findings provide long-overdue experimental justification for applying this phenotype approach on three important crop species, representing the major monocots and dicot groups as well as C_4_ and C_3_ photosynthesis. The lack of large effects due to excision, as well as the occurrence of some small but significant effects, are discussed in the following sections.

### Stabilized leaf water potential and ABA accumulation underlie the utility of phenotyping photosynthesis in excised leaves

The most important crop species for staple foods are herbaceous annuals, and a key anatomical difference between woody and herbaceous species is the composition of the xylem tissue. Primary xylem is the only form of xylem for most herbaceous plants, including cereals (Růžička *et al.*, 2015), although tomato can demonstrate a small amount of secondary xylem (Qi *et al.*, 2020). While primary xylem comprises a relatively thin central zone within stems and leaves, secondary xylem grows out and differentiates from the vascular cambium (Kirst *et al.*, 2004). Secondary xylem serves multiple purposes beyond facilitating water transport, including biomechanical support and protection against physical damage (Sperry, 2003; Morris *et al.*, 2016; Miodek *et al.*, 2021). Avoiding xylem damage is critical for maintaining hydraulic conductivity and protecting photosynthesis, and breakage of the water column through xylem cavitation can disrupt this. Our results are supportive of the notion that the transpiration stream of the excised tomato, barley, and maize leaves was maintained following recutting them under water as we did not observe negative effects on leaf water status (measured as leaf water potential; Fig. 1). The utility of performing photosynthetic measurements on excised leaves from woody species might be supported by the presence of secondary xylem tissue, since such measurements are typically performed by excising the woody branches attached to the leaves of interest. Furthermore, vulnerability segmentation work has shown that branches are more resistant to xylem cavitation than terminal leaves (Zhu *et al.*, 2016). Stem and leaf hydraulic conductance are coordinated (Pivovaroff *et al.*, 2014), and therefore excising leaves might remove the association of leaf and stem hydraulic conductance. It is possible that the reduction in leaf water potential that we observed in barley leaves that had remained attached to the plant (Fig. 1B) was a function of this, and that the process of detaching the leaf from the stem and immersing it in water increased water availability by removing the hydraulic resistance imposed by the stem. Alternatively, the observed difference might suggest that the plants attached to the measured leaves were marginally water stressed; however, this is unlikely since we ensured that all plants were well-watered throughout their growth and on the day before measurements, and the absolute water potential values were higher than –0.25 MPa. Moreover, the light-saturated gas exchange data for barley did not demonstrate any signal of water stress in the attached leaves relative to the excised leaves (as discussed below). Overall, the values for water potential and the minor difference between the excised and attached leaves suggests a lack of water stress.

It has been suggested that roots are essential for stimulating ABA biosynthesis in leaves (Takahashi *et al.*, 2018); however, many studies that have involved excised leaves have demonstrated that ABA levels can increase independently of roots (Bauer *et al.*, 2013; Sussmilch *et al.*, 2017; McAdam and Brodribb, 2018). It is therefore possible that any treatment effects observed as a result of leaf excision could be due to foliar ABA accumulation. Depending on the site and method of excision, the act of cutting a leaf or a tiller from a plant can elicit a wounding-type response, and ABA could accumulate in response as a regulator of downstream defense mechanisms (Cui *et al.*, 2013; Savatin *et al.*, 2014). In addition, the process of excision is likely to affect xylem tension and thus influence the flow rate of water to the leaf. This might also result in ABA accumulation in the leaf to stabilize leaf water status by reducing transpiration; however, no evidence presently exists to support this notion. Moreover, it is likely that the subsequent recutting of leaves underwater, as per this study and other studies, will quickly re-establish xylem tension by mitigating against cavitation. The role of ABA in regulating ion transport into guard cells to induce stomatal closure is well characterized (Schroeder *et al.*, 2001), and hence if leaf excision were to induce leaf ABA accumulation through any mechanism, we would expect to see associated reductions in gas exchange as result of stomatal closure. In this regard, our results demonstrated that in the morning following leaf excision there was no significant difference in the leaf ABA concentration between the attached and excised leaves in any of the three species (Fig. 2). Performing leaf excision on the day prior to phenotyping was carried out because we hypothesized that this would allow sufficient time for degradation of any additionally accumulated ABA. So, while our results do not allow us to determine whether excision stimulated an immediate ABA response, they are supportive of a lack of differential accumulation of ABA in the leaves that were excised on the day prior to measurements. Consequently, we recommend adopting this approach when phenotyping excised leaves to avoid any confounding effects that may potentially arise due to ABA accumulation.

### Differences between excised and attached leaves for gas-exchange traits are limited, but important and species-specific

Our methodology of leaf excision mirrored a widespread approach that has been utilized by many research groups for performing photosynthesis-related phenotyping of material that is difficult to measure *in situ* for a multitude of reasons. This approach is a practical necessary as it enables the large-scale screening and characterization of important diversity within crop species. However, leaf excision will represent a significant shock to the leaf and is likely to elicit substantial wounding responses. To date, few data have existed to support the efficiency of this approach for generating results that match those obtained from leaves that are still attached to the plant. Perhaps the most important finding of our study is that we did not observe a significant effect of leaf excision on light-saturated *A*_N_. This was true for all three species tested and across the morning and afternoon measurements (Fig. 3A, E, I), and goes a long way to verifying the utility of this approach for assessing light-saturated photosynthesis at ambient CO_2_. While there were no significant treatment effects on *A*_N_, we did observe a significant effect of time of measurement for barley and maize, with lower values being recorded in the afternoon. This is indicative of the diurnal pattern of photosynthesis in many species, where there is a decline in *A*_N_ in the afternoon (Singsaas *et al.*, 2000; Mohotti and Lawlor, 2002; Ainsworth *et al.*, 2007; Koester *et al.*, 2016; Vialet-Chabrand *et al.*, 2017). This can be attributed to multiple factors, including declining electron transport and changes to the apparent sink strength due to increasing leaf carbohydrate content (Singsaas *et al.*, 2000; Koester *et al.*, 2016). Hence, while the excision method is supportive of reliably phenotyping *A*_N_, our findings suggest that interpreting and comparing results obtained across an extended diurnal period should be approached with caution regardless of whether they are collected from attached or excised leaves.

A significant and curious time-of-day effect was detected for *c*_i_ in tomato (Fig. 3C), with increased values in the afternoon for both treatments despite there being no significant changes in *A*_N_ or *g*_s_ that could account for it (Fig. 3A, B). One potential explanation is that it could have been a function of lower Rubisco activity, which was suggested by declining values of *V*_cmax_ for both treatments in the afternoon (Fig. 4C). Lower carboxylation by Rubisco might lead to an increase in CO_2_ in the intracellular air space of the leaf in the afternoon compared to the morning, especially since *g*_s_ was not observed to change across these time periods. This highlights the importance of quantifying photosynthetic capacity to generate a broader picture of the photo-physiological state of the leaf, as opposed to relying on light-saturated measurements made under ambient CO_2_. The decrease in *V*_cmax_ in the afternoon for tomato might have been a function of the diurnal expression patterns of Rubisco activase, which is the molecular chaperone that defines Rubisco activity, and its expression has been demonstrated to decline significantly in the afternoon (Perdomo *et al.*, 2021). The afternoon decline in iWUE in tomato (Fig. 3D) mirrored the increase in *c*_i_ (Leakey *et al.*, 2019) and was a function of the combination of the decline in *A*_N_ (Fig. 3B) and increase in *g*_*s*_ (Fig. 3C), despite the changes it those parameters being non-significant individually.

One of the more surprising results from our study was that maize was the only one of the three species where we detected a treatment effect on *g*_s_, with the excision process resulting in a significant decrease compared to attached leaves (Fig. 3J). This was somewhat surprising since maize leaves are larger and appear more physically robust than barley leaves and the petiole of tomato leaves; however, the result was consistent across the two time-points, so leaf excision clearly impaired stomatal opening in maize. The C_4_ carbon-concentrating mechanism that defines the relative de-coupling of *g*_s_ and *A*_N_ in C_4_ species relative to C_3_ species (Leakey *et al.*, 2019) might explain why we did not observe a concurrent treatment effect on *A*_N_ in maize. Thus, the decrease in *g*_s_ observed in the excised leaves was not sufficient to decrease *c*_i_ enough to lose CO_2_ saturation of *A*_N_ (Fig. 3K), as evidenced by the *A*_N_/*c*_i_ responses (Fig. 4I, J). The treatment effects of leaf excision in maize on *g*_s_ but not on *A*_N_ led to expected results for *c*_i_, namely that it decreased significantly at both time-points due to the decreased capacity for CO_2_ uptake (Fig. 3K). Concurrently, the stomatal resistance to water loss was greater, such that iWUE was higher in excised leaves at both time-points and particularly so in the afternoon (Fig. 3L). Taken together, these results show that the time-of-day effects on light-saturated rate of gas exchange were greater in attached maize leaves, thus highlighting the potential for longer measurement days when working with excised leaves instead.

Despite the differences in maize described above, we did not observe any significant effects in terms of treatment or time on the photosynthetic capacity traits *V*_pmax_ and *A*_max_ (Fig. 4K, L). This suggests that the reduced *g*_*s*_ at the starting point of the *A*_N_/*c*_i_ response measurements (i.e. at ambient CO_2_) in the excised leaves did not compromise the ability to assess the rate of photosynthetic induction from low-to-ambient CO_2_ concentrations or the rates of photosynthesis when *g*_s_ was minimal at high CO_2_ concentrations. Our results are therefore supportive of performing *A*_N_/*c*_i_ response curves in excised maize leaves to model photosynthetic capacity. In general, our results were also supportive of performing such response curves on excised leaves of the C_3_ species. We did detect a significant reduction in *V*_cmax_ in excised leaves of barley (Fig. 4G), but the difference was relatively minor and was not exaggerated by an interaction with time.

### Reflectance and chlorophyll fluorescence appear relatively unchanged between attached and excised leaves

There were no significant differences detected for leaf reflectance between the treatments for any of the species on a wavelength-by-wavelength basis (Fig. 5). However, the patterns of *P*-values across the spectrum were surprisingly unique between the species, and were different between the time-points for barley. In general, if we consider the dominant factors that influence leaf reflectance (Kokaly *et al.*, 2003), the patterns of *P*-values suggest that the most excision-sensitive aspects of reflectance were due to leaf water content in tomato and light-scattering in the mesophyll in barley and maize. Our results from barley and maize are similar to those previously observed by Richardson and Berlyn, (2002) across certain tree species, where it was suggested that reductions in the near-infra red spectra reflectance in excised leaves were related to leaf structural changes at the cellular level. Thus, declining leaf water status results in cytoplasmic shrinkage, such that light-scattering is altered at the interface between the cell walls and water (Carter, 1991; Aldakheel and Danson, 1997; Richardson and Berlyn, 2002). However, we did not observe perturbed leaf water status in the excised leaves (Fig. 1), and hence this might explain why the differences in reflectance between the treatments that we found did not pass significance thresholds (Fig. 5). Richardson and Berlyn, (2002) tested differences between excised and attached leaves over several days and they did not begin to detect significant differences in reflectance indices and chlorophyll fluorescence until 48 h after excision. Hence, it is possible that we might have observed differences in reflectance and other traits had we measured for a further day; however, this is not common practice with crop species, and we would advise against it.

To further resolve any potential differences in reflectance between attached and excised leaves, we calculated five commonly employed indices (Supplementary Table S1; Supplementary Figs S3–S5), and we were only able to detect two statistically significant differences. For barley, the moisture stress index was significantly higher in the morning in excised leaves, suggesting a reduction in leaf water content; however, this effect was not apparent in the afternoon (Supplementary Fig. S4D). In maize, a significant treatment effect for the Datt index, which is positively correlated to chlorophyll content (Lu *et al.*, 2015), suggested that the content was reduced in excised leaves (Supplementary Fig. S5B). It could be hypothesized that ABA accumulation might be the underlying mechanism resulting in this difference, since there is some evidence linking leaf yellowing and reductions in leaf chlorophyll to ABA; however, that was evidently not the situation in our study (Fig. 2) and this is typically a longer-term effect (Wang *et al.*, 2018). In general, our results suggested that changes in reflectance between the excised and attached leaves were slight, and this is similar to what has previously been shown in woody species (Richardson and Berlyn, 2002).

Our results also mirrored those of Richardson and Berlyn, (2002) in that we saw no differences in *F*_v_/*F*_m_ between attached and excise leaves, irrespective of the system used to obtain the results (Fig. 6; Supplementary Fig. S6). This demonstrated that electron flow in PSII was not significantly affected by leaf excision, and hence there was no impairment of the photosynthetic apparatus. We also found no significant differences in the dynamics of NPQ, except for a minor reduction in the rate of induction in excised tomato leaves when measured using the gas-exchange leaf chamber fluorometer (Supplementary Fig. S6B). No such difference was detected the following day using the closed chlorophyll fluorescence system (Fig. 6B), suggesting that it probably resulted from insufficient time allowed for dark adaption, especially since the *F*_v_/*F*_m_ value was also bordering on being significantly reduced in excised leaves when measured in the leaf chamber fluorometer (*P*=0.07; Supplementary Fig. S6A).

### Conclusions

Screening large amounts of material for diversity in photosynthesis-related traits is only realistically possible with excised leaves, and hence our results are important in validating this approach in terms of comparison with leaves that remain attached to the plant. However, our study does highlight the existence of specifies-specific treatment effects that researchers should be aware of, for example a reduction in *g*_s_ in maize. This should encourage those who are planning to adopt this phenotyping approach to validate whether the technique is suitable for their species of interest. One salient point that our study does not address is the potential for differential responses to excision on an intraspecific basis. An obvious further extension to this study would be to test how genetically distinct genotypes within a species perform following leaf excision, as this has the potential to have important consequences for studies focusing on cultivar improvements within a particular crop.

## Supplementary data

The following supplementary data are available at *JXB* online.

Fig. S1. Overview of the experimental workflow.

Fig. S2. Mean hyperspectral reflectance data collected in the morning and afternoon.

Fig. S3. Derived hyperspectral reflectance indices for tomato.

Fig. S4. Derived hyperspectral reflectance indices for barley.

Fig. S5. Derived hyperspectral reflectance indices for maize.

Fig. S6. Variation in traits associated with chlorophyll fluorescence between excised and attached leaves measured using the leaf chamber fluorometer.

Table S1. Reflectance indices used in this study.

erad319_suppl_Supplementary_Table_S1_Figures_S1-S6

## Data Availability

All data supporting the findings of this study are available within the paper and within its supplementary data published online.

## Abbreviations

ABA: abscisic acid
*A*_max_: maximum rate of photosynthesis
*A*_N_: net photosynthetic CO_2_ assimilation
*c*_i_: intracellular CO_2_ concentration
*F*_v_/*F*_m_: maximum photosystem II efficiency
*g*_s_: stomatal conductance
iWUE: intrinsic water use efficiency
*J*_max_: maximum rate of electron transport for RuBP regeneration
NPQ: non-photochemical quenching
PAR: photosynthetically active radiation
*V*_cmax_: maximum rate of Rubisco carboxylation
*V*_pmax_: maximum rate of PEPc carboxylation
