# Supplementary material for: Excised leaves show limited and species-specific effects on photosynthetic parameters across crop functional types
Source: J Exp Bot. 2023 Aug 11;74(21):6662–76. doi: 10.1093/jxb/erad319 (PMC10662226; doi:10.1093/jxb/erad319)
Supplement: erad319_suppl_Supplementary_Table_S1_Figures_S1-S6 [file erad319_suppl_supplementary_table_s1_figures_s1-s6.pdf]

Supplemental Table S1. Reflectance indices used in this study

Reflectance Indices	Equation	Reference
Red Edge Normalised Difference Vegetation Index (RE-NDVI)	$(750_R-705_R)/(750_R+705_R)$	He *et a*l (2020) Scientific Reports
Datt index	$((719_R-726_R)/719_R-743_R)$	Datt (1999) Journal of Plant Physiology
Normalised Difference Nitrogen Index (NDNI)	$(\log(1/1510_R)-\log(1/1680_R))/(\log(1/1510_R)+\log(1/1680_R))$	Wang *et al* (2016) Remote Sensing
Moisture Stress Index (MSI)	$1600_R/820_R$	Hunt *et al* (1989) Remote Sensing of the Environment
Leaf Water Index (LWI)	$1300_R/1450_R$	Seelig *et al* (2009) Irrigation Science

References

Datt .1999. A New reflectance Index for Remote Sensing of Chlorophyll Content in Higher Plants: Test using *Eucalyptus* leaves. *Journal of Plant Physiology* 154 (1): 30-36.

He L, Ren X, Wang Y, Liu B, Zhang H, Liu W, Feng W, Guo T. 2020. Comparing methods for estimating leaf area index by multi-angular remote sensing in winter wheat. *Scientific Reports* 2020; 10: 13943

Wang Z, Wang T, Darvishzadeh R, Skidmore AK, Jones S, Suarez L, Woodgate W, Heiden U, Heurich M, Hearne J. 2016. Vegetation Indices for Mapping Canopy Foliar Nitrogen in a Mixed Temperate Forest. Remote Sensing 2016 8 (6): 491

Hunt ER, Rock BN. 1989. Detection of changes in leaf water content using Near- and Middle-Infrared reflectances. *Remote Sensing of Environment* 30 (1) 43-54.

Seelig HD, Hoehn A, Stodieck LS, Klaus DM, Adams III WW, Emer WJ. 2009. Plant water parameters and the remote sensing R_{1300}/R_{1450} leaf water index: controlled condition dynamics during the development of water deficit stress. *Irrigation Science* 27: 357-365.

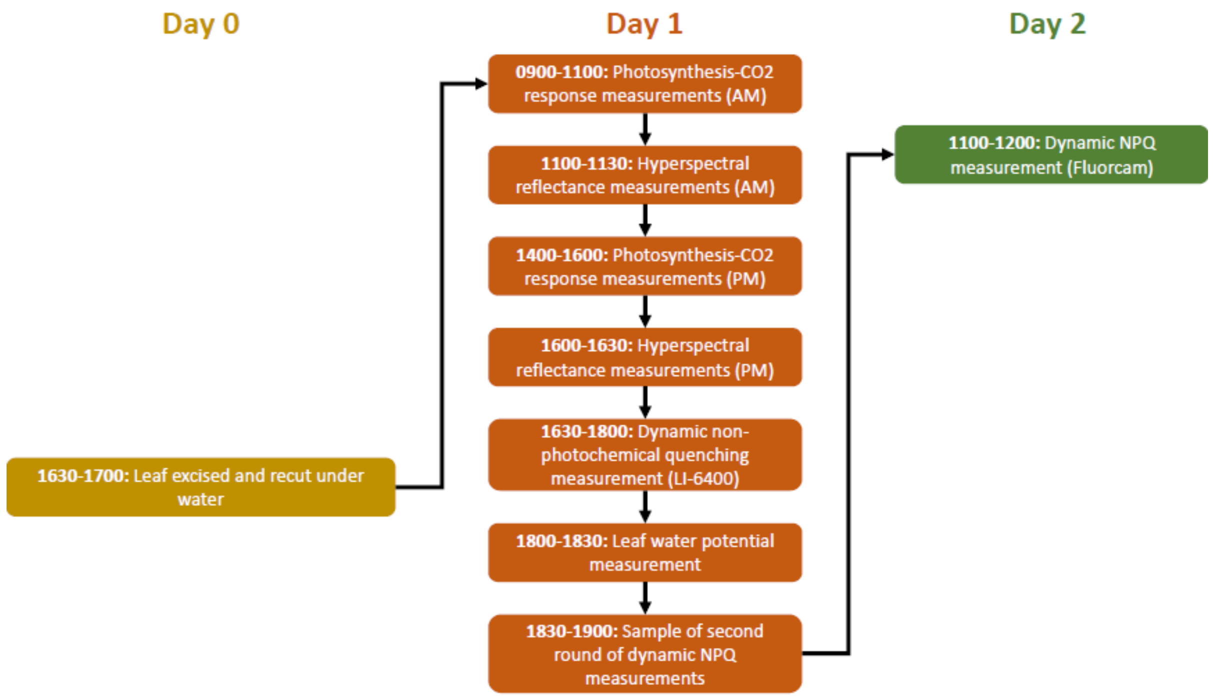

Supplemental Figure S1. Overview of experimental workflow.

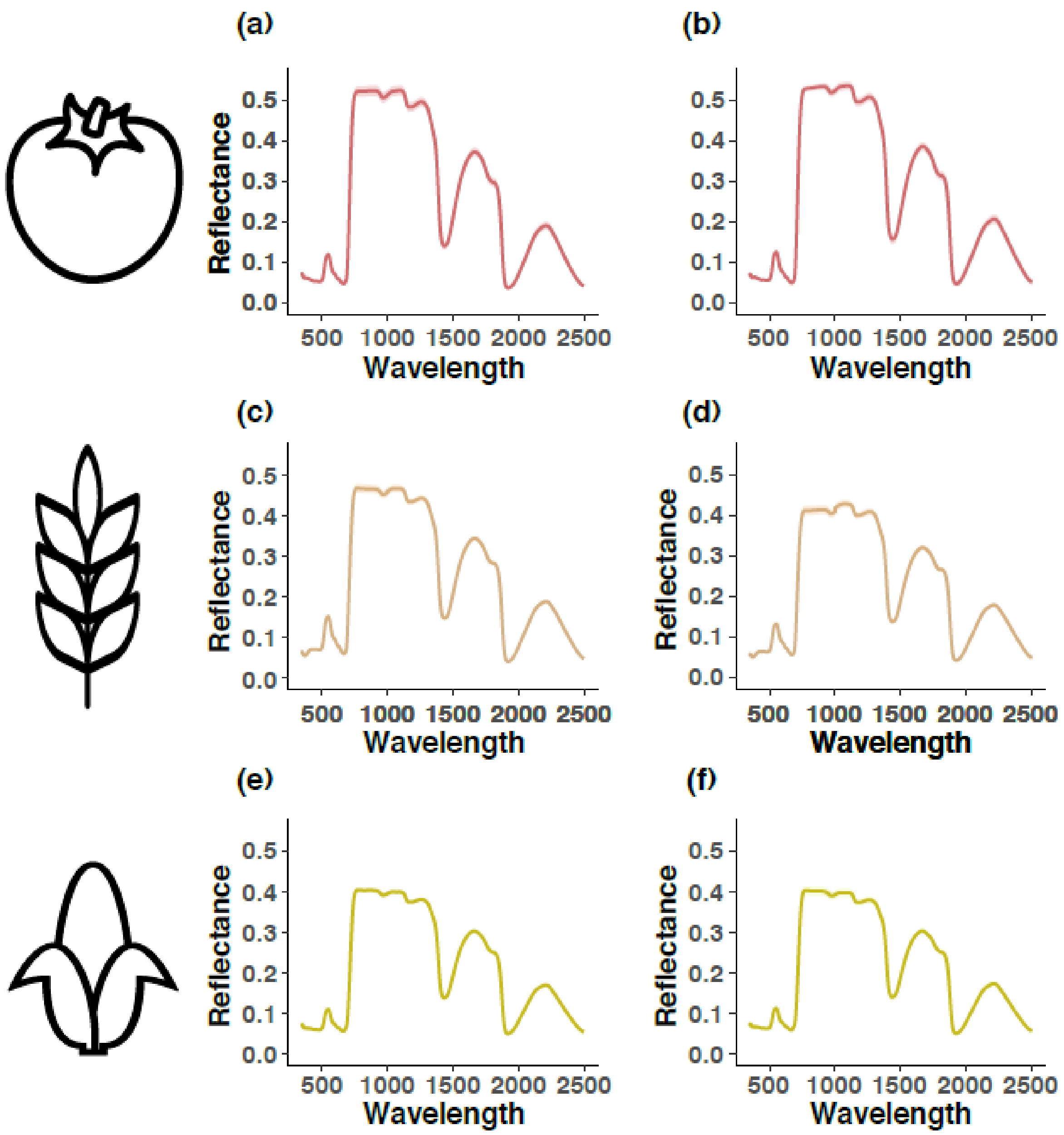

Supplemental Figure S2. Mean hyperspectral reflectance data. The solid line represents the mean reflectance, and the shaded area represents the standard error of the mean. (a) Tomato AM (b) Tomato PM (c) Barley AM (d) Barley PM (e) Maize AM (f) Maize PM

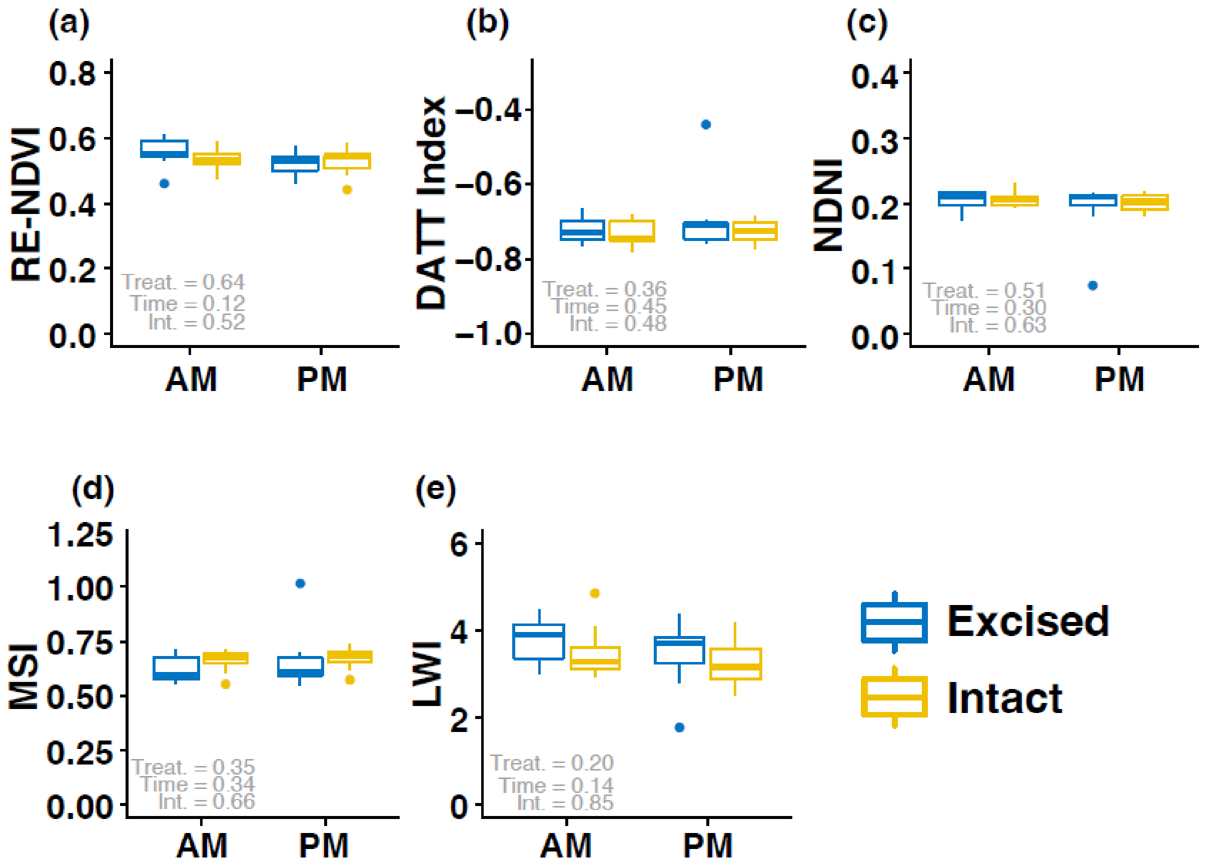

Supplemental Figure S3. Hyperspectral reflectance derived indices for tomato. (a) Red Edge-Normalised Difference Vegetation Index (RE-NDVI), (b) Datt Index (c) Normalised Difference Nitrogen Index (NDNI), (d) Moisture Stress Index (MSI), and (e) Leaf Water Index (LWI)

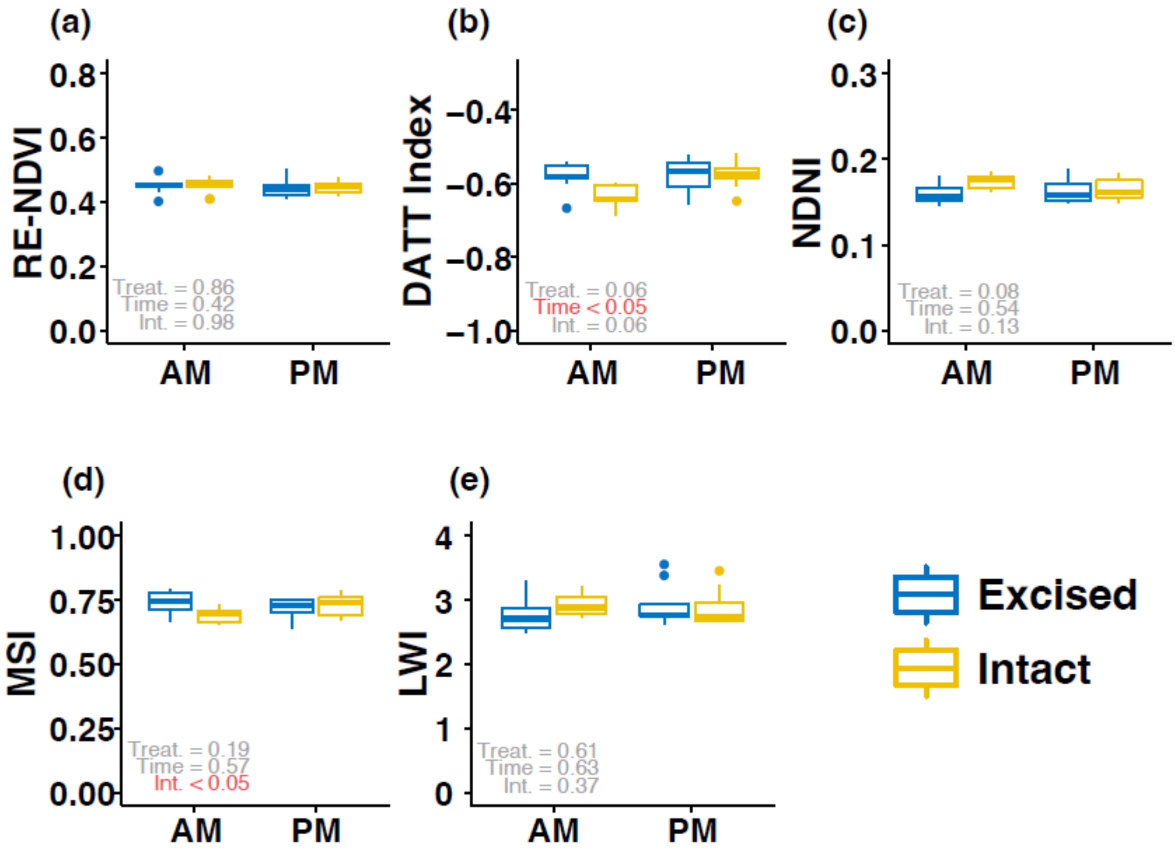

Supplemental Figure S4. Hyperspectral reflectance derived indices for barley. (a) Red Edge-Normalised Difference Vegetation Index (RE-NDVI), (b) DATT Index (c) Normalised Difference Nitrogen Index (NDNI), (d) Moisture Stress Index (MSI), and (e) Leaf Water Index (LWI)

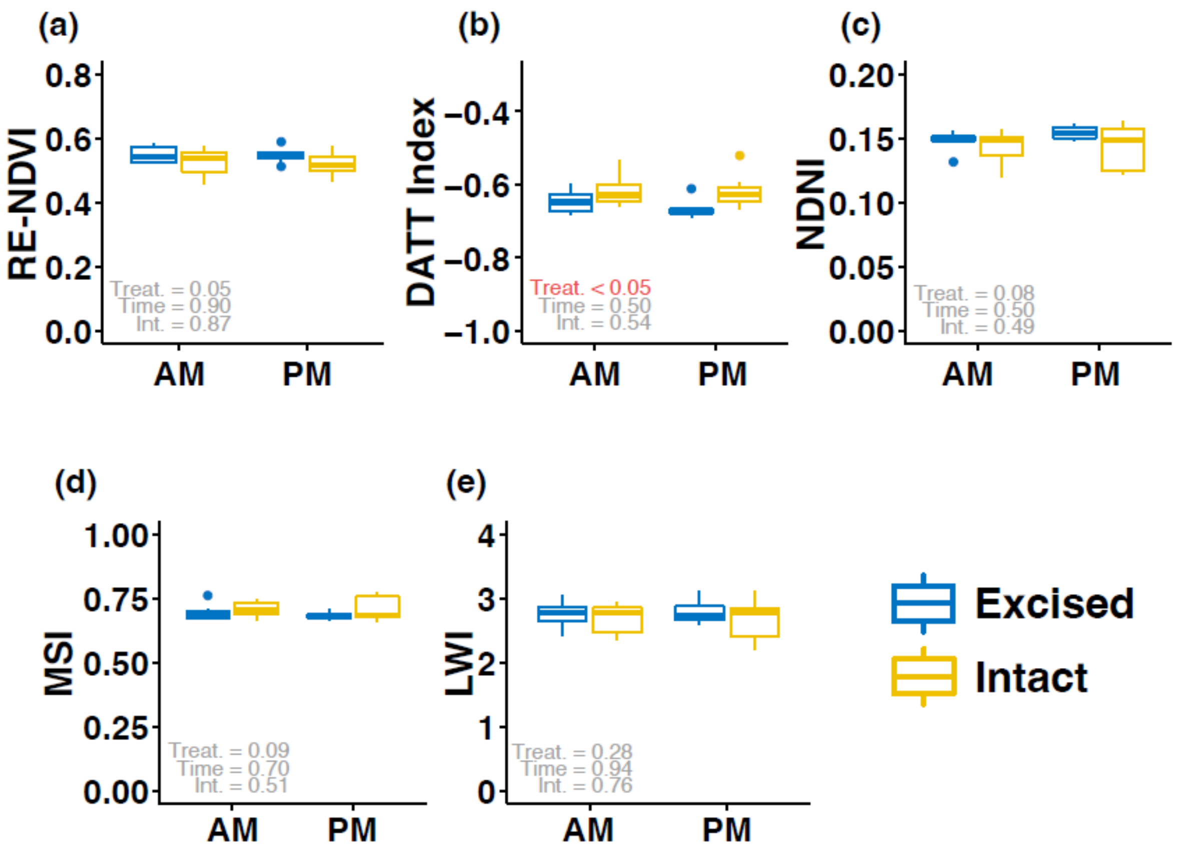

Supplemental Figure S5. Hyperspectral reflectance derived indices for maize. (a) Red Edge-Normalised Difference Vegetation Index (RE-NDVI), (b) DATT Index (c) Normalised Difference Nitrogen Index (NDNI), (d) Moisture Stress Index (MSI), and (e) Leaf Water Index (LWI)

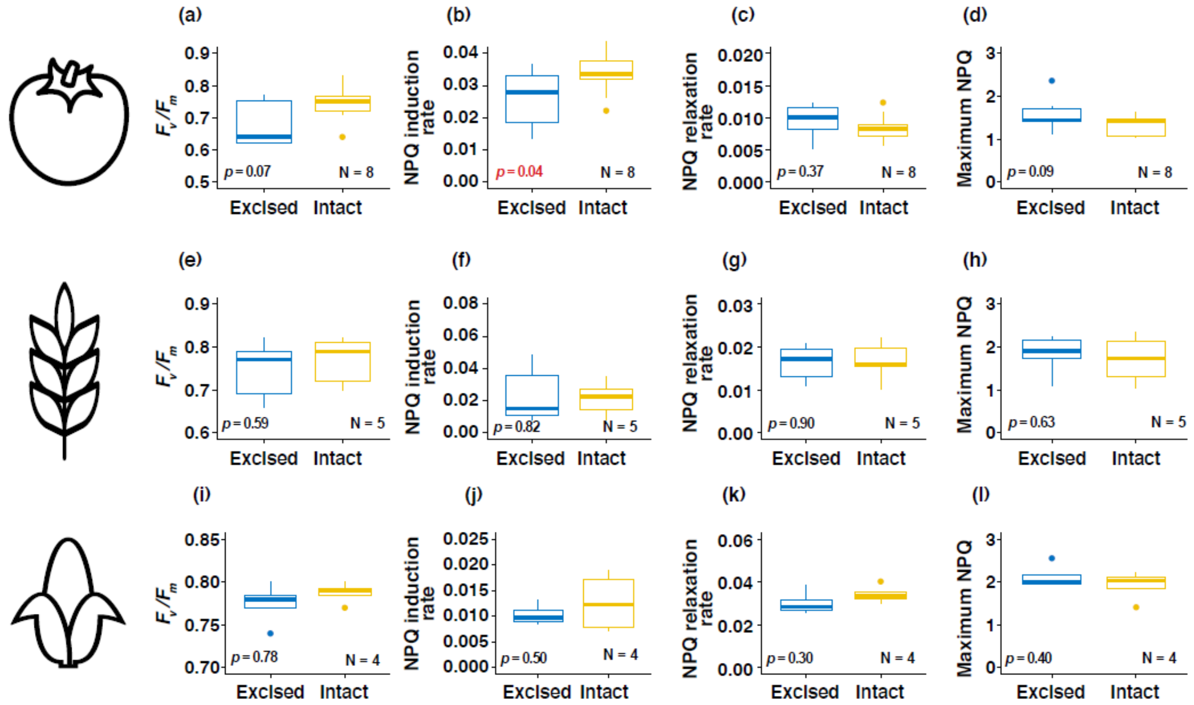

Supplemental Figure S6. Boxplots showing variation in chlorophyll fluorescence associated traits between excised and intact leaves measured using the leaf chamber fluorometer.
